# Impact of African swine fever emergency on the mental health of first responders in the Dominican Republic

**DOI:** 10.1371/journal.pone.0342159

**Published:** 2026-02-03

**Authors:** Rachel A. Schambow, Alejandro D. Perez, Raysa E. R. Santiago, Laura V. Alarcón, Amit K. Pradhananga, Andres M. Perez

**Affiliations:** 1 Center for Animal Health and Food Safety, College of Veterinary Medicine, University of Minnesota, Saint Paul, Minnesota, United States of America; 2 Doctor Armando Zamudio Hospital, Comandante Luis Piedrabuena, Argentina; 3 Instituto de Enfermedades Infecciosas y Zoonóticas, Facultad de Ciencias Agronómicas y Veterinarias, Universidad Autónoma de Santo Domingo, Santo Domingo, Dominican Republic; 4 Facultad de Ciencias Veterinarias, Universidad Nacional de La Plata, La Plata, Buenos Aires, Argentina; 5 Center for Changing Landscapes, University of Minnesota, Saint Paul, Minnesota, United States of America; 6 Department of Veterinary Population Medicine, College of Veterinary Medicine, University of Minnesota, Saint Paul, Minnesota, United States of America; Zambia Ministry of Health, ZAMBIA

## Abstract

African swine fever (ASF) has become a global pandemic, affecting nearly 2 million pigs since 2022 alone and causing significant disruption to food security and trade. The Dominican Republic (DR) has been affected by ASF since 2021. Veterinarians are key first responders in animal health emergencies and at risk for negative impacts from prolonged emergency response. We used mixed methods and network analysis to characterize the mental and social impacts of ASF on 29 DR swine veterinarians. All 29 veterinarians were involved with the ASF response through field-based or non-field-based roles. Responses were gathered via a questionnaire and analyzed using network analysis. Preliminary quantitative results were explored further through qualitative focus group interviews. Veterinarians experienced high levels of anger and hopelessness from the ASF epidemic, which were centrally located variables in the network. They were associated with a perceived lack of communication, trust, and transparency between government authorities, veterinarians, and farmers. The impact that animal diseases have on the mental health of veterinarians is often neglected. As animal, human, and zoonotic diseases, such as ASF and avian influenza, continue to emerge and expand, these results bring attention to the need for considering actions to prevent and mitigate their impact on the mental health of first responders and, ultimately, improve the effectiveness of the response.

## Introduction

The ongoing spread of African swine fever (ASF) has become one of the greatest crises for the global animal food industry [1]. ASF is caused by the ASF virus (ASFV), which only infects domestic and wild pigs, does not infect humans, and does not pose a food safety threat. The virus causes a hemorrhagic syndrome in infected pigs, resulting in upwards of 100% mortality in affected herds [1,2]. There is no treatment or widely approved vaccine for ASF. The virus spreads through contact with infected pigs or contaminated objects, and in particular has spread long distances through movements of contaminated people and fomites, and through garbage feeding of infected pork scraps [1,2]. Consequently, since 2007, ASF has spread across 5 world regions and over 60 countries [3]. Since 2022 alone, an estimated 2 million pigs have been lost to ASF or from preventive culling, resulting in major economic losses to affected pig farmers and the global economy [3].

In 2021, ASF was detected for the first time in 40 years in the Americas, affecting almost simultaneously Haiti and the Dominican Republic (DR) [4]. Together, the DR and Haiti make up the island of Hispaniola, which was previously affected by ASF in the 1970s [5]. In 1978 and with the support of their militaries, both countries launched a joint campaign to cull all swine on the island, which was ultimately successful in eradicating the disease but caused significant socioeconomic impacts, particularly to smallholder producers [6]. The response since 2021 has been centered around depopulation of infected herds to prevent further virus spread, which is considered a standard practice for the control of ASF [7]. While ASF has remained contained without further spread in the Americas, the disease has not yet been eradicated from Hispaniola. Meanwhile, ASF has caused significant impact to the island’s swine industry, which consists mainly of informal and small-scale farmers who use pigs as a source of subsistence and economic value [8]. In the DR, pork production decreased by 15% compared to prior to the ASF epidemic, and $28.4 million USD has been paid out to over 5,000 producers for depopulated farms. For consumers, pork prices have increased 10% since July 2021 [8].

While the animal health and economic impacts of ASF are well-recognized, similarly to other animal health emergencies, the effects on the mental health of first responders have been largely neglected. By comparison, the COVID-19 pandemic has generated much research on the mental health impacts of infectious disease emergencies. The World Health Organization has estimated that, following the COVID-19 pandemic, the impact of anxiety and depression on the human population has increased approximately 25%, with first respondents being more highly impacted than the average of the population [9–11]. Similarly to these human healthcare professionals, the mental health of veterinarians is affected during animal health crises, given their role as first responders [12,13]. Baysinger and Kogan (2022) observed that swine veterinarians engaged in mass animal depopulation activities due to slaughterhouse infrastructure breakdown in the United States during the COVID-19 pandemic suffered high levels of mental health issues and ethical or moral distress [13]. In extreme circumstances, this emotional distress may even lead to suicidal tendency in veterinarians.

Mental health diagnosis is typically measured through the use of scales or scoring systems. There are multiple challenges associated with the use of these methods. Because setting up appropriate cut-off values is somewhat subjective, the accuracy of scoring systems is often uncertain, which may result in false positive and false negative results [14,15]. Furthermore, the accuracy of these scoring systems is influenced by the context of the targeted population, impairing the ability to apply this methodology broadly [16]. Finally, much of the moral injury research carried out to date has taken place in military contexts. Comparatively, very little research has examined experiences of moral injury in veterinary professionals, especially considering that experience shows mental impairment in first responders in similar previous epidemics [17,18].

The objective of this paper was to explore and describe the impact that ASF has had on the mental health of first respondents in the DR, demonstrating the impact that non-zoonotic diseases have on the health of the public. Additionally, the novel analytical approach followed here, resulting from a combination of mixed qualitative and quantitative methods, could serve as a framework for assessing the impact of diseases on mental health of first respondents, farmers, and relevant stakeholders. The results presented here ultimately help raise awareness and demonstrate the need for designing and implementing policy intended to protect the mental health of veterinarians and first responders in the face of epidemics.

## Methods

### General approach

This protocol was reviewed and approved by the University of Minnesota Institutional Review Board (#00020636). To explore and describe the impacts of ASF on the mental and social health of veterinarians responding to ASF in the DR, a sequential explanatory mixed methods approach was used. During the first stage, individual responses were collected from veterinarians involved in the ASF emergency response using a structured questionnaire and analyzed using a network approach. In the second stage, a semi-structured focus group was used to collect qualitative data based on initial quantitative findings from the questionnaire.

### Study population

The official veterinary services of the DR counts 29 official veterinarians in the ASF control field activities. The vast majority were reassigned to ASF activities from other programs of the Animal Health Department. At the time when the ASF epidemic started, there was no record of private swine veterinarians in the DR [19]. In January 2025, the official veterinary services of the DR, in collaboration with the Universidad Autonoma de Santo Domingo and the University of Minnesota, launched an accreditation program for official and private swine veterinarians in the DR. Because the program offered a certificate free of charge and, according to DR regulations, it was expected that only accredited veterinarians would be authorized to work on swine operations, it was assumed that the cohort of training participants (n = 29 public and private veterinarians) represented much of the population of swine veterinarians in the DR. The study population was comprised of these 29 veterinarians, which served as cases exposed to the ASF emergency response; no unexposed study subjects (controls) were included in the study population.

### Questionnaire development and administration

To gather individual quantitative responses, a questionnaire was developed to assess components of physical, mental, and social well-being following the ASF outbreaks in the DR (S1 File). Questions were adapted from the World Health Organization Division of Mental Health Quality of Life assessment [20]. A mixture of multiple choice, multiple option, and short answer questions were used. The questionnaire was written and administered in Spanish via the online platform Qualtrics.

Prior to participation, all participants were verbally advised of the study objectives, the reason for the study, how their data would be handled, and the potential risks of participation. Participation in this activity was not a mandatory part of the training workshop and was entirely voluntary. All participants reviewed and signed a written consent form, in conjunction with the requirements of the University of Minnesota Institutional Review Board. Responses were collected anonymously with no identifying information.

On January 17, 2025, the questionnaire was individually and anonymously completed by 29 veterinarians at the in-person workshop under the facilitation of the co-authors. The questionnaire data were analyzed using R version 4.4.2 in RStudio version 2024.12.1 and Microsoft Excel version 365 version 2501. Frequency tables were produced that summarized the count and percentage for each categorical response. The number of negative mental or social signs reported per individual was calculated.

### Network analysis

Network analysis is a useful and powerful tool for describing and analyzing systems-level problems, allowing for visualization and quantitative characterizations of complex and interdependent systems [21]. Networks are constructed of a subject or variable of interest, represented as nodes, and the interactions or connections between them, represented as lines (edges) connecting the nodes. Networks may be directed, whereby connections have uni- or bidirectionality from one node to another implying a directional link or movement, or undirected, implying no specific direction of the connections between nodes. Some relevant network terms and metrics are defined in Table 1. Previously, network analysis has been used to study social contacts between people and animals (social network analysis) [22], disease transmission modeling (transmission or contact networks) [23], and opinions and beliefs (attitude networks) [24], amongst many others examples from numerous fields and disciplines. Network analysis is a useful framework for examining and understanding the complex relationships between mental and social health symptoms, as has been previously proposed [25]. Generally, this approach suggests that these symptoms are causally linked and that their relationships can be encoded in a network structure.

**Table 1:** Description of network analysis terminology and metrics. Definitions adapted from Saqr et al., (2024).

Term	Definition
Node	The subject or unit of interest for the network, such as a person, farm, or response variable.
Edge	The unique connection between two nodes, which may represent interactions, movements, associations, or other links between them.
Edge density	The proportion of edges present of all possible edges between nodes in the network.
Diameter	The longest geodesic distance (length of the shortest path between two nodes) in the network.
Mean Distance	Average shortest path length between nodes.
Degree	The number of unique edges per node.
Average degree	Average number of edges per node in the network.
Betweenness	Node-level score based on the number of geodesics (shortest paths) going through a given node. Higher values indicate a node is located on the shortest path between many other nodes. May be normalized by the number of node pairs in the network.
Closeness	Node-level score based on the number of steps that are required to access every other node from a given node. Estimated as the inverse of distance, such that higher values indicate increased closeness. May be normalized using the inverse average distance to all reachable nodes.
Eigenvector	Node-level score that reflects the importance of a node based on the centralities of nodes it is connected to. It is based on the connectivity of a given node to other nodes, those nodes’ connectivity to other nodes, and so on. Higher values indicate higher connectivity.
Eccentricity	Node-level distance to the farthest other nodes in the network. Higher values indicate farther distances.
Transitivity (Global Clustering Coefficient)	Graph-level probability that adjacent nodes of a given node are connected. Higher values indicate higher clustering.
Centralization	Graph-level estimation of the extent to which the network is organized around a few nodes. Can be based on degree, betweenness, or closeness
Global Efficiency	Graph-level measurement of the effectiveness of the network structure as a conduit for information exchange using the distances between vertices. Higher values indicate increased efficiency.

Here, a network approach was used to understand the relationships between the mental and social response variables reported by DR veterinarians responding to ASF. Preliminary networks were constructed to help guide the development of questions for qualitative data collection. Networks were constructed to visualize responses with the highest overall and positive agreement, respectively. Two-by-two contingency tables were used to estimate agreement between variables. All variables were coded as the increased or more negative impact level = 1, and the decreased or less negative impact = 2. Variables with more than 2 categories were dichotomized (S1 Table). For each pair, the overall proportion of agreement (OPA) and positive proportion of agreement (PPA) were calculated. OPA was calculated as the sum of concordant responses (i.e., cells 1,1 and 2,2 of the contingency table) divided by the total number of responses. PPA was calculated as the number of positive/affirmative concordant responses (i.e., cell 1,1) divided by the total number of responses. The top 25% of values (High OPA: ≥ 0.793 [maximum = 0.996]; High PPA: ≥ 0.138 [maximum = 0.379]) were selected for inclusion in the networks. Questionnaire response variables were represented as nodes, and agreement was represented as edges in the networks. The strength of the OPA or PPA value was represented by increased line thickness and darker color of edges. Separate undirected networks were built based on OPA and PPA connections.

Network-level and node-level characteristics were estimated using the *igraph* package in R v4.4.2 and visualized using the *ggraph* and *ggplot2* packages. Network size was characterized by estimating the number of nodes, number of edges, diameter, and mean distance. Network connectivity metrics were estimated including edge density, average degree, transitivity, and global efficiency. Network centralization was estimated using degree centralization, betweenness centralization, and closeness centralization. Node-level characteristics metrics that were estimated included degree, normalized betweenness, normalized closeness, Eigenvector, and eccentricity.

Sensitivity analysis was used to explore the stability of the network structure and centrality metrics given the small sample size. A simulation-based method was used in a manner similar to previous literature [26]. For each of the OPA and PPA networks, a random subset of 10, 20, 30, 40, and 50% of the agreement values (OPA or PPA) were replaced with a randomly generated proportion to simulate “rewiring” of the network, with equal probability of any value being selected between 0–1. This was repeated for 1,000 iterations for each level of randomness (10–50%). Then, for each iteration, networks were constructed and analyzed in the same fashion as previously described. For each level of randomness, average network figures were produced by calculating the proportion of times a node or edge appeared in the network, and mean and standard deviation of node-level centrality metrics were calculated.

### Qualitative focus group and qualitative data analysis

To validate and explore the questionnaire findings, semi-structured focus groups were used with the same veterinarian participants at a second training workshop held in Santo Domingo, DR in March 2025. All veterinarians that responded to the questionnaire were present at the second workshop. Prior to the workshop, guiding questions were developed based on the preliminary network analysis of mental and social health variables to guide the discussion (S2 File).

The focus group was held on March 22, 2025. Before starting the focus group discussion, the participants were again asked to review and accept a written consent form. All 29 participants completed the form. The activity was organized in three stages. In the first stage, preliminary results from the questionnaire and network analysis were shown to the participating veterinarians. Then, in the second stage, participants were split into two groups, one consisting of government veterinarians and one consisting of private, practicing veterinarians. Each group was facilitated by a moderator and an assistant moderator, all of whom were native Spanish speakers. During the discussion, each group was first asked to express their agreement or disagreement with any of the preliminary findings that were shown to them. Then, the groups were asked to discuss aspects of the ASF response that contributed to feelings of anger, frustration, hopelessness, or sadness, given these responses’ centrality in the PPA network. Finally, participants were asked to discuss aspects that contributed to feeling positive about the future or helped to alleviate stress and suffering during the outbreaks. In the third stage of the activity, the groups were asked to interact with each other to promote further understanding and exploration of each group’s perspective during the ASF outbreaks. To do so, each group was asked to report a summary of their discussion to the other group. Then, the second group repeated back the key messages they heard. This was repeated with both groups. Collectively, all participants identified points of agreement between them and areas for future collaboration.

Notes taken by the assistant moderators during the discussion were used for the analysis. A thematic analysis approach was used to analyze the qualitative data [27]. First, the data were coded using an inductive coding method, whereby codes emerge from data itself, as opposed to having a predetermined list of codes. Initial analysis was performed by two individuals, including one native Spanish speaker. Codes were reviewed, then grouped into subthemes and themes. All subthemes and themes were reviewed with the research team.

## Results

### Questionnaire responses

All 29 veterinarians that responded to the questionnaire were involved in the response to ASF. Twelve veterinarians had seen ASF outbreaks in the field (mean = 19 outbreaks per veterinarian, range = 1–80 outbreaks). The most commonly self-reported negative sign since the ASF outbreaks was new feelings of anger or frustration (48%; Table 2) followed by feelings of hopelessness or sadness (41%). More veterinarians who saw ASF outbreaks in the field (67%) reported anger or frustration compared to those who did not (35%). Over a quarter of the veterinarians also reported reduced sleep, reduced energy, or reduced enjoyment of life. Despite reports of negative signs, 86% of veterinarians indicated they felt positive about the future. Considering questions on social health and work, respondents mostly reported no changes (Tables 2 and 3). In the comment field for their behavior toward neighbors, two veterinarians reported that they reduced contact to help prevent disease spread, and one reported that they had experienced increased meetings and events between producers.

**Table 2:** Responses to yes/no questions from the mental and social health questionnaire administered to 29 swine veterinarians in the Dominican Republic, by all respondents, those who saw African swine fever in the field (n = 12), and those who did not (n = 17).

Question	Number of Yes Responses (Percent)		
	All respondents (n = 29)	Saw field outbreaks (n = 12)	Did not see field outbreaks (n = 17)
Do you feel positive about the future?	25 (86.2)	10 (83.3)	15 (88.2)
Have you experienced reduced energy since the ASF outbreak?	8 (27.6)	6 (50)	2 (11.8)
Have you experienced reduced enjoyment of life since the ASF outbreak?	8 (27.6)	4 (33.3)	4 (23.5)
Have you experienced new feelings of anger or frustration since the ASF outbreak?	14 (48.3)	8 (66.7)	6 (35.3)
Have you experienced reduced sleep since the ASF outbreak?	9 (31)	6 (50)	3 (17.6)
Have you experienced new feelings of hopelessness or sadness since the ASF outbreak?	12 (41.4)	6 (50)	6 (35.3)
Do you have trouble concentrating on tasks since the ASF outbreak?	2 (6.9)	1 (8.3)	1 (5.9)
Have you experienced poor memory since the ASF outbreak?	5 (17.2)	3 (25)	2 (11.8)
Have you experienced extreme changes in feelings of happiness and sadness since the ASF outbreak?	7 (24.1)	4 (33.3)	3 (17.6)
Do you have less self-worth or less confidence in yourself due to the ASF outbreak?	5 (17.2)	3 (25)	2 (11.8)
Have you had any intrusive thoughts about death or dying since the ASF outbreak?	2 (6.9)	1 (8.3)	1 (5.9)
Since the ASF outbreak, have you had any intrusive thoughts that your family or community would be improved if you were gone?	1 (3.4)	0 (0)	1 (5.9)
Have you started or increased your visits to a mental health professional since the outbreak?	4 (13.8)	3 (25)	1 (5.9)
Have you experienced any negative behaviors from your neighbors or social circle since the outbreak?	7 (24.1)	4 (33.3)	3 (17.6)
Do you still treat your neighbors the same pre-outbreak and post-outbreak?	24 (82.8)	9 (75)	15 (88.2)
Have you lost work since the outbreak?	2 (6.9)	1 (8.3)	1 (5.9)
Do you need to attend any continuing education courses?	20 (69)	8 (66.7)	12 (70.6)
Have you been able to continue your continuing education courses post-outbreak?	17 of 20 (85)	6 of 8	11 of 12
Do you have school-age children?	11 (37.9)	7 (58.3)	4 (23.5)
Have you had to remove your children from school due to bullying or ostracization due to an ASF outbreak?	0 of 11 (0)	0 of 7	0 of 4
Have you received any governmental involvement (positive or negative) due to the outbreak?	7 (24.1)	6 (50)	1 (5.9)
Have you experienced reduced energy since the ASF outbreak?	8 (27.6)	6 (50)	2 (11.8)

**Table 3:** Responses to categorical questions from the mental and social health questionnaire administered to 29 swine veterinarians in the Dominican Republic, by all respondents, those who saw African swine fever in the field (n = 12), and those who did not (n = 17).

Question *Response Option*	Number of Respondents (Percent)		
	All respondents (n = 29)	Saw field outbreaks (n = 12)	Did not see field outbreaks (n = 17)
**How have your behaviors in society/your community changed since the outbreak(s)?**			
*Less involved*	4 (13.8)	2 (16.7)	2 (11.8)
*No change*	12 (41.4%)	6 (50)	6 (35.3)
*More involved*	11 (37.9)	4 (33.3)	7 (41.2)
*Wasn’t involved*	2 (6.9)	0 (0)	2 (11.8)
**Have you experienced any changes in your physical exercise since the ASF outbreak?**			
*Less*	4 (13.8)	3 (25)	1 (5.9)
*About the same*	17 (58.6)	5 (41.7)	12 (70.6)
*More*	8 (27.6)	4 (33.3)	4 (23.5)
**Is your physical health better or worse since the ASF outbreak?**			
*Worse*	8 (27.6)	6 (50)	2 (11.8)
*No Change*	20 (69)	6 (50)	14 (82.4)
*Better*	1 (3.4)	0 (0)	1 (5.9)
**Has the ASF outbreak caused adverse physical health or emotional problems that have made it difficult for you to do social activities?**			
*No*	7 (24.1)	4 (33.3)	3 (17.6)
*No Change*	17 (58.6)	4 (33.3)	13 (76.5)
*Yes*	5 (17.2)	4 (33.3)	1 (5.9)
**How many farms did you visit per day pre-outbreak?**			
*Less than 1*	19 (65.5)	8 (66.7)	11 (64.7)
*1–3*	8 (27.6)	2 (16.7)	6 (35.3)
*4–5*	1 (3.4)	1 (8.3)	0 (0)
*More than 5*	1 (3.4)	1 (8.3)	0 (0)
**How many farms do you visit per day post-outbreak?**			
*Less than 1*	19 (65.5)	4 (33.3)	15 (88.2)
*1–3*	5 (17.2)	4 (33.3)	1 (5.9)
*4–5*	3 (10.3)	2 (16.7)	1 (5.9)
*More than 5*	2 (6.9)	2 (16.7)	0 (0)
**How has the time you spend working changed since the outbreak?**			
*Much less*	2 (6.9)	1 (8.3)	1 (5.9)
*Somewhat less*	2 (6.9)	1 (8.3)	1 (5.9)
*About the same*	13 (44.8)	2 (16.7)	11 (64.7)
*Somewhat more*	4 (13.8)	3 (25)	1 (5.9)
*Much more*	8 (27.6)	5 (41.7)	3 (17.6)
**How has your job satisfaction changed since the outbreak?**			
*Much worse*	4 (13.8)	3 (25)	1 (5.9)
*Somewhat worse*	2 (6.9)	0 (0)	2 (11.8)
*About the same*	15 (51.7)	4 (33.3)	11 (64.7)
*Somewhat better*	5 (17.2)	3 (25)	2 (11.8)
*Much better*	3 (10.3)	2 (16.7)	1 (5.9)
**What kind of involvement (financial/quarantine/fines/etc? Check all that apply.**			
*Fines*	1 of 7	0 of 6	1 of 1
*Financial Compensation*	5 of 7	4 of 6	1 of 1
*Quarantine and/or isolation*	4 of 7	3 of 6	1 of 1
*Required by government to participate in depopulation on one of the farms I serve*	2 of 7	2 of 6	0 of 1
**Was the involvement overall helpful or harmful to you/the community?**			
*Harmful*	0 of 7	0	0
*Helpful*	7 of 7 (100)	6 of 6	1 of 1

The median number of negative responses per individual was 2, and it ranged from zero to 15, out of 22 total possible negative responses Five veterinarians indicated no negative responses, all of whom reported they did not see any field outbreaks of ASF. Four individuals (14%) reported 11 or more negative responses. All four of these individuals reported that they had experienced worse physical health, reduced sleep, reduced enjoyment of life, poor memory, new feelings of anger and frustration, new feelings of hopelessness and sadness, and having started mental health visits. The individual with the highest score also reported the most severe signs: intrusive thoughts about death or dying, intrusive thoughts that their family or community would be better if they were gone, and that they did not feel positive for the future. This individual did not see any field outbreaks of ASF.

## Network analysis

**Fig 1:**
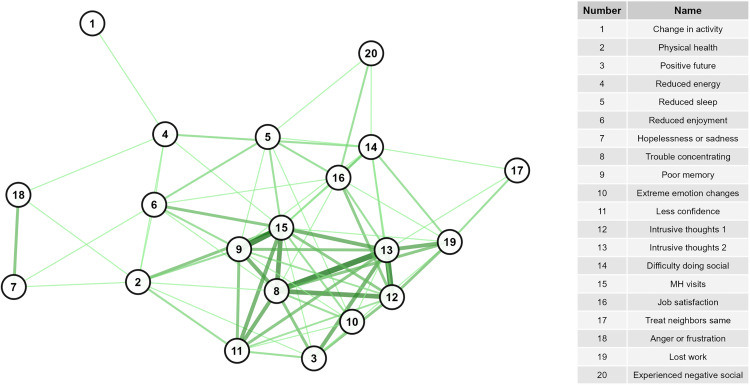
Network of signs with highest levels of overall agreement. Darker line color indicates higher values of overall agreement.

**Fig 2:**
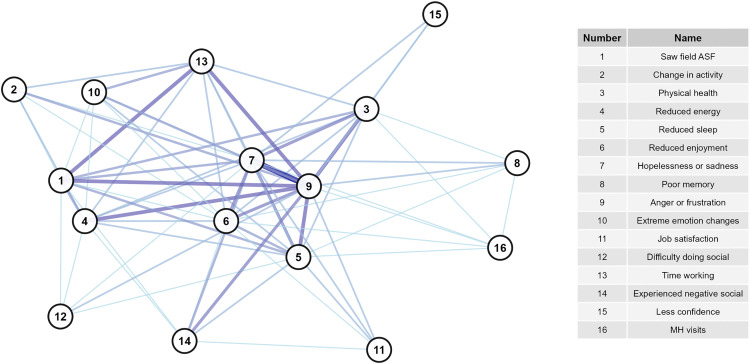
Network of signs with highest levels of positive agreement. Darker line color indicates higher values of positive agreement.

The high OPA network was larger, consisting of 20 nodes (mental health signs) and 73 edges (overall agreement) with a diameter of 4 (Fig 1, S2 Table), whereas the high PPA network was slightly smaller, consisting of 16 nodes and 71 edges (positive agreement) with a diameter of 2 (Fig 2, S2 Table). Mean distance was similar between the OPA (1.79) and PPA network (1.41). Fourteen nodes were the same between both networks. Intrusive thoughts about death and dying, intrusive thoughts about being gone, lost work, feeling positive for the future, treating neighbors the same, and trouble concentrating were unique to the OPA network. Having attended ASF outbreaks in the field and time spent working were unique to the PPA network.

Overall, nodes in the PPA network were more connected than those in the OPA network. The PPA network had higher edge density (0.59) and average node degree (8.9, range 3–15) than the OPA network (edge density = 0.384, average node degree = 7.3, range 1–14). Global transitivity was higher in the PPA network (0.80) than the OPA network (0.66), though both values indicate high network connectivity. Both networks exhibited low betweenness centralization and moderate degree and closeness centralization, indicating that overall, neither network was highly organized around specific nodes.

**Fig 3:**
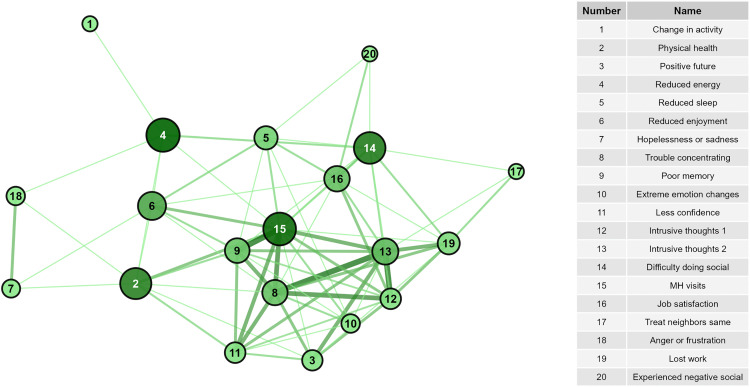
Network of signs with highest levels of overall agreement and node betweenness. Darker line color indicates higher values of overall agreement. Increased node size and darker node color indicates greater betweenness of the node.

**Fig 4:**
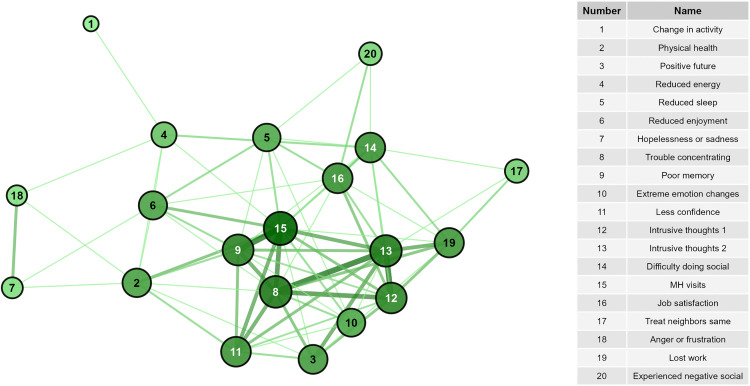
Network of signs with highest levels of overall agreement and node eigenvector. Darker line color indicates higher values of overall agreement. Increased node size and darker node color indicates greater eigenvector of the node.

**Fig 5:**
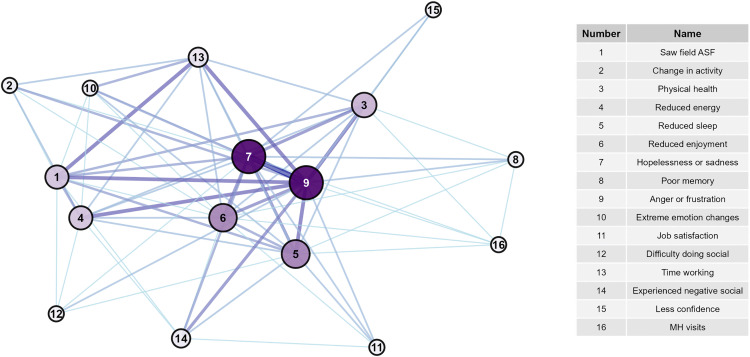
Network of signs with highest levels of positive agreement and node betweenness. Darker line color indicates higher values of positive agreement. Increased node size and darker node color indicates greater betweenness of the node.

**Fig 6:**
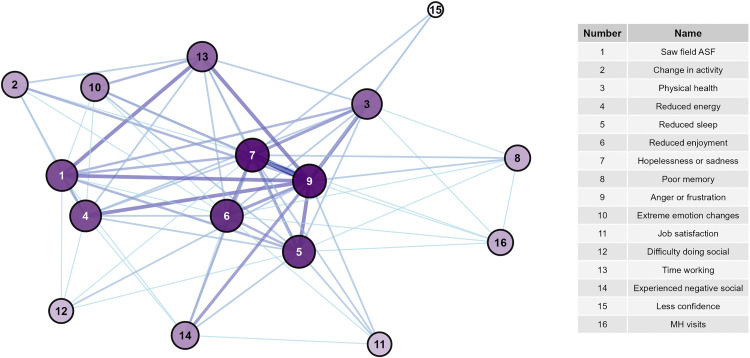
Network of signs with highest levels of positive agreement and node eigenvector. Darker line color indicates higher values of positive agreement. Increased node size and darker node color indicates greater eigenvector of the node.

Considering node-level centrality, in the OPA network, starting mental health visits was the most central (Figs 3 and 4, S3 Table) with the highest degree, closeness, and Eigenvector scores. Other highly central nodes included intrusive thoughts about being gone, trouble concentrating, reduced energy, and difficulty doing social activities. Treating neighbors the same and hopelessness/sadness had the highest eccentricity scores, though the range of eccentricity was low (2–4) given the network’s high connectivity. In the PPA network, hopelessness or sadness, anger or frustration, reduced enjoyment of life, and reduced sleep had the highest degree, betweenness, closeness, and Eigenvector scores, placing them more central in the network compared to other nodes (Figs 5 and 6, S3 Table). Anger and hopelessness/sadness had the lowest eccentricity scores (1), while the remaining nodes all had scores of 2, consistent with the overall high connectivity of the network.

The sensitivity analysis suggests that generally the OPA network is more robust than the PPA network, with node-level centrality metrics remaining stable and more similar to their original values across larger values of simulated data (S3 File, S4 File). Inferences from the PPA network stayed similar at 10 and 20% levels of randomness, while network structure generally dissolved at 40 and 50% levels. Overall, main conclusions for each network stayed similar: anger and hopelessness were central variables in the PPA network, which was generally smaller than the OPA network; and mental health visits were most central within the OPA network.

### Qualitative findings

**Fig 7:**
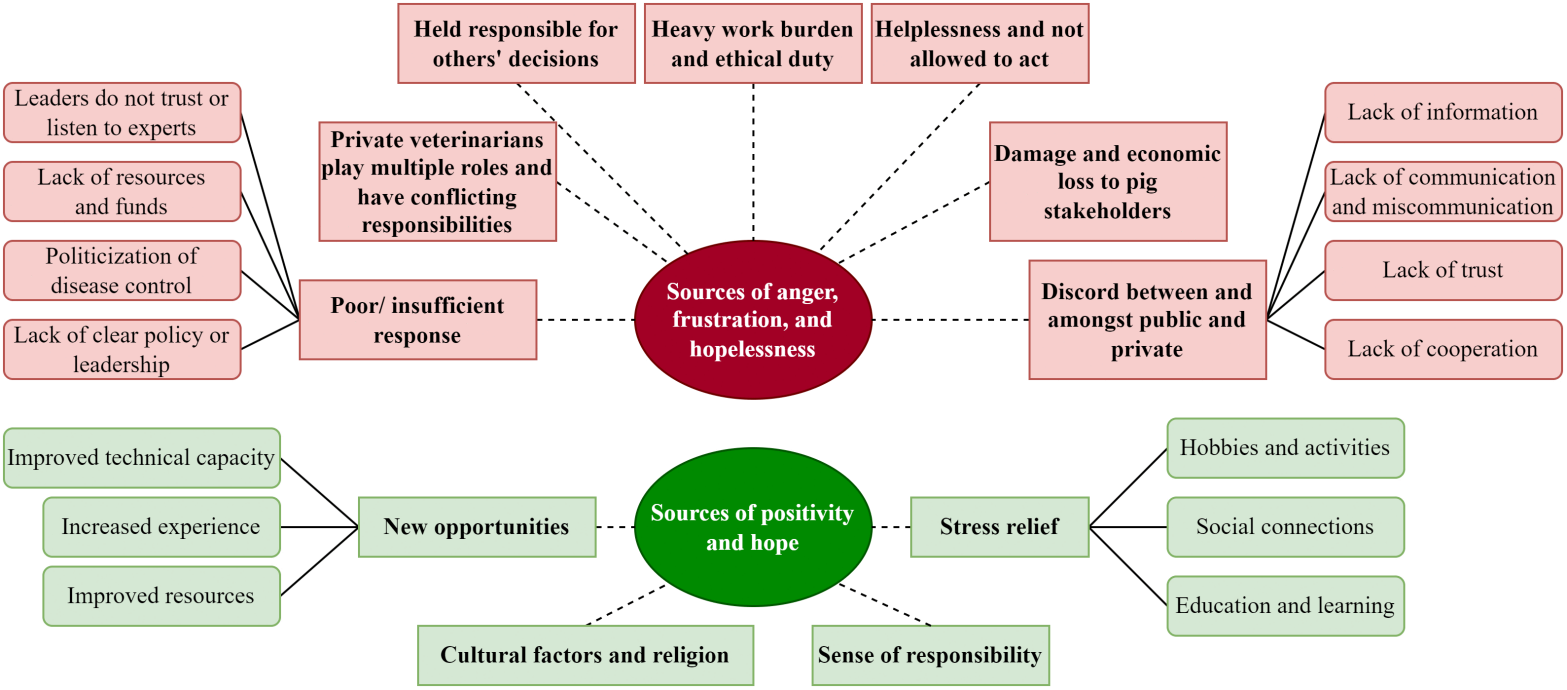
Map of focus group themes and subthemes. Themes depicted as rectangular boxes, and subthemes depicted as rounded rectangular boxes, regarding sources of anger, frustration and hopelessness, and sources of positivity and hope, as discussed during the focus group interview with 29 veterinarians in the Dominican Republic.

Seven main themes emerged from the discussions surrounding sources of anger, frustration, and hopelessness (Fig 7). The private veterinarians indicated that they felt frustration and anger because the authorities hid the truth of the initial diagnosis of the disease and because they made decisions slowly and based on politics, causing spread of ASF. They often felt unable to act, leading to feelings of frustration, anger, and powerlessness. These issues generated division, mistrust, and competition between the governmental and non-governmental sectors. One participant expressed, while another nodded in agreement:

*We worked face to face with the government vet when the sampling started, and we decided that the private vet would take the samples to prevent people from entering the farm. It was very criticized, and they closed doors on us. For example, they would not give us permits to move animals for a month, this increased the anger, the powerlessness, the resentment that continues. I still haven’t talked to some government officials. (Translated from anonymous)*

Many of the private veterinarians commented that they felt psychological pressure and responsibility to the farmer, as the farmer demanded that they constantly provide updated ASF information and prevent the entry of the disease to their farm, but there were no resources to invest in biosecurity. If the farm became infected, veterinarians felt pressured by farmers not to report, but felt conflicted about their professional values and ethical duty to prevent ASF spread. Two participants expressed:

*Producers ask many questions, and you don’t find answers with the authorities. They ask you not to say anything about the disease, but you feel obliged to notify them; you can’t hide it. They are given a deadline to inform officially. Otherwise, you look bad as a veterinarian, and it would be professional suicide and generate a double burden, powerlessness, and anxiety. (Translated from anonymous)*

Participants, with angry and resigned expressions, commented that the situation worsened because the community was misinformed. Because of media publications about the ASF, some stopped eating national pork. This affected them economically, as fewer pigs were sold and fewer veterinary products were produced. Consequently, they indicated that this negative situation was damaging older people’s retirement and discouraging younger generations of veterinarians from becoming involved in the pig sector.

Participants from the government group commented that they felt frustrated because there was no clear policy, little to no resources, and no organized plan. One participant commented: *“When it was mentioned that there was an outbreak, it was chaos, and no one knew what to do”.* Many participants reported they felt angry because technical experts were removed from their positions and not listened to, despite their training in disease management. They believe that this resulted in a waste of available resources. Two participants expressed anger, sadness, and guilt about not having the resources to carry out culling activities and not getting answers when producers asked for financial compensation, as it took several months for them to be paid. They identified that there was a lack of trust and cooperation between public and private stakeholders. Many government participants said it was frustrating not to be paid for all the extra work they had done. In addition to all the frustration, sadness, and stress felt from slaughtering sows on farms and slaughterhouses, they were not provided with basic necessities such as food and water during long working hours. Several participants agreed that the work had to be done anyway, out of vocation and professional ethics. A common statement expressed was: *“The official veterinarian has to do it and always be available”*.

Four main themes emerged from the discussion of sources of positivity and hope (Fig 7). Participants from both groups expressed that a sense of responsibility to help the situation kept them going, and that their sources of support or relief were doing sports or recreational activities, traveling, enjoying time with their family and colleagues, or diversifying their learning into other species or diseases, such as poultry farming. Both groups highlighted many new opportunities to improve control of ASF. The group of private veterinarians expressed that the most important finding was that despite frustration, anger, and powerlessness, they feel hopeful because they believe they are able and willing to overcome the disease. Many participants were optimistic regarding the improved capacity of the official diagnostic laboratory and the improved biosecurity of the farms. Veterinarians in the government group expressed that they were happy and somewhat relieved that, finally, the official service was in charge of the control program and that they could collaborate more with the private sector. One veterinarian commented that the lesson is: “*The state alone cannot do it; the producers alone cannot do it*.” They identified positive aspects including an improved technical approach, new proposals and ideas, and experience gained over the previous few years.

## Discussion

This study represents one of the few to explore and describe the effects of prolonged emergency response on veterinary first responders using a novel mixture of network analysis and qualitative methods. Often, non-zoonotic disease emergencies are solely viewed as animal health or economic emergencies. However, these findings reveal the damaging mental health and social impacts experienced by veterinarians that have been responding to ASF over the past 4 years, and they highlight the need to prepare veterinary first responders to handle difficult situations and intense stress. Ultimately, this may improve the quality of the response against animal health emergencies such as ASF.

The veterinarians here most commonly reported experiencing anger and frustration, hopelessness and sadness, reduced sleep, reduced energy, and reduced enjoyment of life. These findings are consistent with previous studies of veterinary first responders in the Philippines, United Kingdom, and Japan during animal health emergencies, and amongst first responders and human health care workers during the COVID-19 pandemic [17,28–31]. The qualitative focus groups revealed that work overload, scarcity or mismanagement of resources, miscommunication, constant exposure to animal death, and loss of economic livelihood for producers generated anger, guilt, and heavy burdens for both governmental and non-governmental veterinarians. Despite the many negative signs reported, the majority of veterinarians felt positive for the future. Compared to the beginning of the epidemic, they felt they were strengthened in their technical training, experience, diagnostic capacity and speed, and had opportunities for improved collaboration between public and private stakeholders.

These results provide evidence of the intense professional and institutional demands placed upon veterinary first responders, giving rise to symptoms of anger, frustration, and sadness, many of which persist today. They support the need for preparedness efforts in ‘peace time’ to develop and review policy and legislation for emergency animal disease response and to build intersectoral collaboration and trust amongst public and private stakeholders. Importantly, the current results do not support a difference in the severity of impacts between those who saw and did not see ASF in the field, nor do they suggest that one experience is inherently more negatively impactful than another. For example, the highest number of negative signs was experienced by someone who did not see ASF in the field, highlighting that veterinarians in non-field roles also experienced high levels of distress. The qualitative findings suggest that factors such as administrative burden, unclear communication, or perceived lack of recognition could be critical drivers of this distress. Veterinarians in both office and field-based roles during emergency response need preparedness and support for potential negative mental health impacts.

The network of high positive agreement (PPA, Fig 2) helps to describe the interconnectedness of the negative signs experienced by the most impacted veterinarians. Hopelessness/sadness and anger/frustration were central variables to this network. Because of their high connectivity and centrality, it appears that these feelings have the capacity to modulate many other signs and behaviors if they are not appropriately addressed. Comparatively, the network of overall agreement (OPA, Fig 1) reflects connections for both those who did and did not report higher levels of negative impacts. This network may suggest that as the levels of particular variables increase or decrease, the connected nodes will respond in a similar fashion. In this network, mental health visits are most centrally located, while hopelessness/sadness and anger/frustration become less centralized. Given that few veterinarians reported starting mental health visits (n = 4), this may suggest that for those who did not report many negative signs, they also did not feel a level of distress that required them to seek professional care.

The network analyses and mixed methods approach used here reveal the strong interconnectedness of the negative mental and social health impacts and provides a novel framework for examining their effects amongst veterinary first responders. The concept that mental health clinical signs interact in a network structure and are not independent is a developing area in psychology and psychopathology [32]. Using this framework, interventions can be developed that target both the symptoms and the relationships between symptoms. Practically, this can include mentally and emotionally preparing veterinarians for how they may feel during the emergency response, such as powerlessness, anger, or hopelessness, and equipping them with techniques to help them manage stress and negative emotions.

This study had some limitations given the study design used. Within a cross-sectional study, it is not possible to determine the causal and temporal links between signs; for example, one cannot tell whether anger preceded hopelessness, or vice versa. Without the presence of a control group, it is difficult to fully attribute the source of negative effects to the ASF outbreaks. Future studies should strive to use study designs that incorporate a control group and allow for more precise and exact estimation of risk and impacts. However, the definition of exposure and selection of control subjects should be carefully designed to provide a control group similar across important covariates to cases [33]. In particular, defining exposure versus non-exposure to the ASF emergency will require careful consideration that incorporates cultural and country-specific factors. Collecting information on subjects’ duties and roles, and experiences will help to define exposure status, and will allow for more critical examination of how these relate to potential negative impacts that might be experienced during the response. In a similar manner, demographic information, which were not collected here, should also be collected.

Regarding the use of network analysis, it is not well known yet how well the group-level findings of network structures of mental health signs predict individual-level variance. In particular here, the small sample size makes extensive use of quantitative techniques like network analysis difficult, as centrality measures may be unstable and strongly influenced by outliers or by additional data. Recall and reporting bias may also be present; for example, the four individuals experiencing the highest number of signs all had started mental health care. It may be possible that these individuals are more aware of their emotions, and consequently, more able and willing to report them. This potential bias reinforces the need to provide veterinarians with appropriate training so that they can recognize and address their own mental health needs, and to normalize help-seeking behaviors.

## Conclusions

This study describes the negative mental health and social impacts that veterinarians experienced as a result of responding to the ASF epidemic in the Dominican Republic. This work highlights the critical need for specific training to prepare veterinary first responders for the range of stressful situations they will encounter during animal disease emergencies to help protect their mental health and well-being, whether they work primarily in the field or in office-based roles. Ultimately, by strengthening the resiliency of veterinary first responders, we may improve the quality of the response to these societal threats.

## Supporting information

S1 File Mental and social health questionnaire administered to veterinarians in the Dominican Republic, as translated to English (original version administered in Spanish). (PDF)

S2 File Pre-developed guiding questions for focus group discussions with 29 veterinarians in the Dominican Republic regarding their experiences as responders during the African swine fever outbreaks. (PDF)

S3 File Average networks and node-level degree and betweenness of OPA and PPA networks, for sensitivity analysis using 10, 20, 30, 40, and 50% levels of randomized data. (PDF)

S4 File Average centrality metrics from sensitivity analysis of OPA and PPA networks, for sensitivity analysis using 10, 20, 30, 40, and 50% levels of randomized data. (XLSX)

S1 Table Dichotomization and leveling of variables for network analysis. (PDF)

S2 Table Global-level network measurements of High OPA and High PPA networks. (PDF)

S3 Table Local node-level centrality measures for nodes in high OPA and high PPA networks. *NA = Not applicable to this network.
(XLSX)
