# Supplementary material for: Impact of African swine fever emergency on the mental health of first responders in the Dominican Republic
Source: PLoS One. 2026 Feb 3;21(2):e0342159. doi: 10.1371/journal.pone.0342159 (PMC12867258; doi:10.1371/journal.pone.0342159)
Supplement: S1 File — (PDF) [file pone.0342159.s001.pdf]

Supplementary File 1. Mental and social health questionnaire administered to veterinarians in the Dominican Republic, as translated to English (original version administered in Spanish).

In the following section, we aim to collect information about the public health impacts of ASF outbreaks on physical, mental and social wellbeing.

Physical Wellbeing: How are veterinarians, producers, and consumers affected by a non-zoonotic animal disease outbreak?

Mental & Behavioral Wellbeing: How has mental and behavioral health been impacted since a non-zoonotic animal disease outbreaks has occurred?

Social Wellbeing: How has society been impacted by the non-zoonotic animal disease outbreak?

Would you like to answer this section from your perspective as a veterinarian or farmer/producer? Please choose the most applicable role for you.

o Veterinarian

o Producer

Veterinarians only: Have you attended field outbreaks of ASF?

o Yes

o No

Veterinarians only: How many field outbreaks of ASF have you attended?

____

Physical Wellbeing

In the next section, we ask you to consider the impacts of ASF on your physical wellbeing.

Is your physical health better or worse since the ASF outbreak?

o Better

o Worse

o No change

Have you experienced any changes in your physical exercise since the ASF outbreak?

o Exercise more

o Exercise less

o Exercise about the same

Mental and Behavioral Wellbeing

In the next section, we ask you to consider the impacts of ASF on mental, behavioral, and emotional wellbeing.

Have you experienced reduced energy since the ASF outbreak?

o Yes

o No

Have you experienced reduced sleep since the ASF outbreak?

o Yes

o No

Have you experienced reduced enjoyment of life since the ASF outbreak?

o Yes

o No

Do you feel positive about the future?

o Yes

o No

Have you experienced new feelings of hopelessness or sadness since the ASF outbreak?

o Yes

o No

Do you have trouble concentrating on tasks since the ASF outbreak?

o Yes

o No

Have you experienced poor memory since the ASF outbreak?

o Yes

o No

Have you experienced new feelings of anger or frustration since the ASF outbreak?

o Yes

o No

Have you experienced extreme changes in feelings of happiness and sadness since the ASF outbreak?

o Yes

o No

Do you have less self-worth or less confidence in yourself due to the ASF outbreak?

o Yes

o No

Have you had any intrusive thoughts about death or dying since the ASF outbreak?

o Yes

o No

Since the ASF outbreak, have you had any intrusive thoughts that your family or community would be improved if you were gone?

o Yes

o No

Has the ASF outbreak caused adverse physical health or emotional problems that have made it difficult for you to do social activities (such as visiting to friends or family)?

o Yes

o No

o No change

Have you started or increased your visits to a mental health professional since the outbreak?

o Yes

o No

Mental and Behavioral Wellbeing: Work and Employment

How many farms did you visit per day before the ASF outbreak?

o Less than 1

o 1-3

o 4-5

o More than 5

How many farms do you visit per day after the outbreak?

o Less than 1

o 1-3

o 4-5

o More than 5

Have you lost work since the outbreak?

o Yes

o No

How has the time you spend working changed since the outbreak?

o Much less

o Somewhat less

o About the same

o Somewhat more

o Much more

How has your job satisfaction changed since the outbreak?

o Much worse

o Somewhat worse

o About the same

o Somewhat better

o Much better

Social Wellbeing

In this final section, we ask you to consider topics related to your social wellbeing before and after an ASF outbreak.

Veterinarians only: Do you need to attend any Continuing Education courses?

o Yes

o No

Veterinarians only: Have you been able to continue those continuing education courses after the ASF outbreak?

o Yes

o No

Do you have school-age children?

o Yes

o No

If you have children, have you had to remove your children from school due to bullying or ostracization due to an ASF outbreak?

o Yes

o No

Have you experienced any negative behaviors from your neighbors or social circle since the outbreak?

o Yes

o No

What types of negative behaviors are your neighbors showing you and why do you think they are acting this way?

____

How have your behaviors in society/your community changed since the outbreak(s)?

o More involved in community

o Less involved in community

o No change to community involvement

o Was never involved in community

Do you still treat your neighbors the same pre-outbreak and post-outbreak?

o Yes

o No

What has changed, and why?

____

Have you received any governmental involvement (positive or negative) due to the outbreak?

o Yes

o No

What kind of involvement (financial/quarantine/fines/etc)? Check all that apply.

- ☐ Financial compensation
- ☐ Fines
- ☐ Quarantine/isolation
- ☐ Required by government to participate in depopulation on one of the farms I serve

Was the involvement overall helpful or harmful to you/the community?

o Helpful

o Harmful
