# Supplementary material for: Impact of African swine fever emergency on the mental health of first responders in the Dominican Republic
Source: PLoS One. 2026 Feb 3;21(2):e0342159. doi: 10.1371/journal.pone.0342159 (PMC12867258; doi:10.1371/journal.pone.0342159)
Supplement: S2 File — (PDF) [file pone.0342159.s002.pdf]

**Supplementary File 2. Pre-developed guiding questions for focus group discussions conducted with 29 veterinarians in the Dominican Republic regarding their experiences as responders during the African swine fever outbreaks.**

1. Do you agree with the results presented?
2. What situations do you highlight as the most important findings?
3. What aspects caught your attention?
4. What things or situations caused you frustration or helplessness? Consider responsibilities with producers, to the community, animal health, farmers, and government.
5. What factors made it (frustration or helplessness) worse?
6. What would you have done differently?
7. What things made the situation difficult?
8. Did you feel heard?
9. Did you find anything positive in the situation? What motivated you to give this answer?
10. What were your sources of support or relief from stress and negative impacts?
