# Supplementary material for: Impact of African swine fever emergency on the mental health of first responders in the Dominican Republic
Source: PLoS One. 2026 Feb 3;21(2):e0342159. doi: 10.1371/journal.pone.0342159 (PMC12867258; doi:10.1371/journal.pone.0342159)
Supplement: S3 File — (PDF) [file pone.0342159.s003.pdf]

Supplementary File 3: Average networks and node-level degree and betweenness of OPA and PPA networks, for sensitivity analysis using 10, 20, 30, 40, and 50% levels of randomized data.

Simulated networks incorporate 10, 20, 30, 40, or 50% simulated randomness in OPA or PPA values for 1,000 iterations each. Network figures depict mean betweenness as the node size, the proportion of iterations that the node appears as node color (darker=higher proportion), and the proportion of iterations that the edge appears as edge color (darker=higher proportion).

Sensitivity Analysis of OPA Network

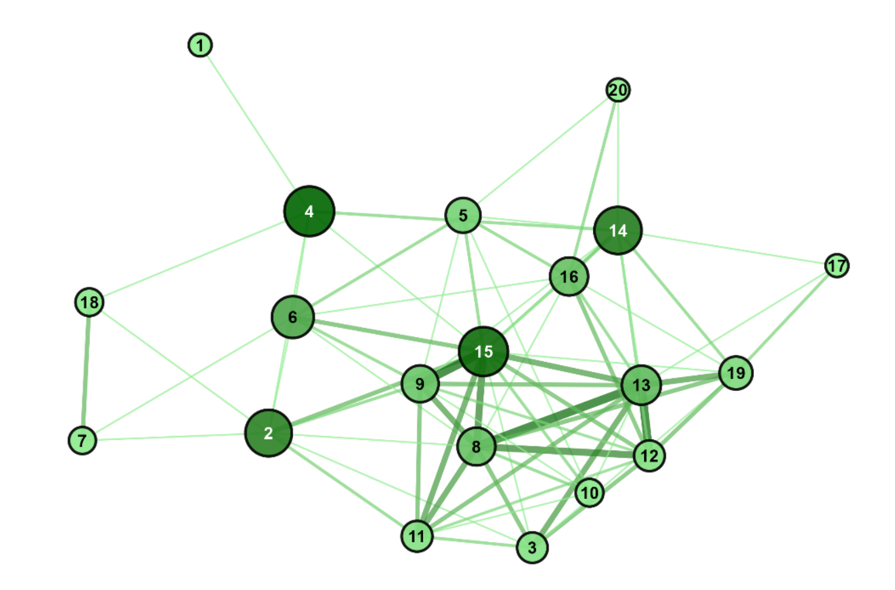

Number	Name
1	Change in activity
2	Physical health
3	Positive future
4	Reduced energy
5	Reduced sleep
6	Reduced enjoyment
7	Hopelessness or sadness
8	Trouble concentrating
9	Poor memory
10	Extreme emotion changes
11	Less confidence
12	Intrusive thoughts 1
13	Intrusive thoughts 2
14	Difficulty doing social
15	MH visits
16	Job satisfaction
17	Treat neighbors same
18	Anger or frustration
19	Lost work
20	Experienced negative social

Original Network (Figure 2a from main manuscript)

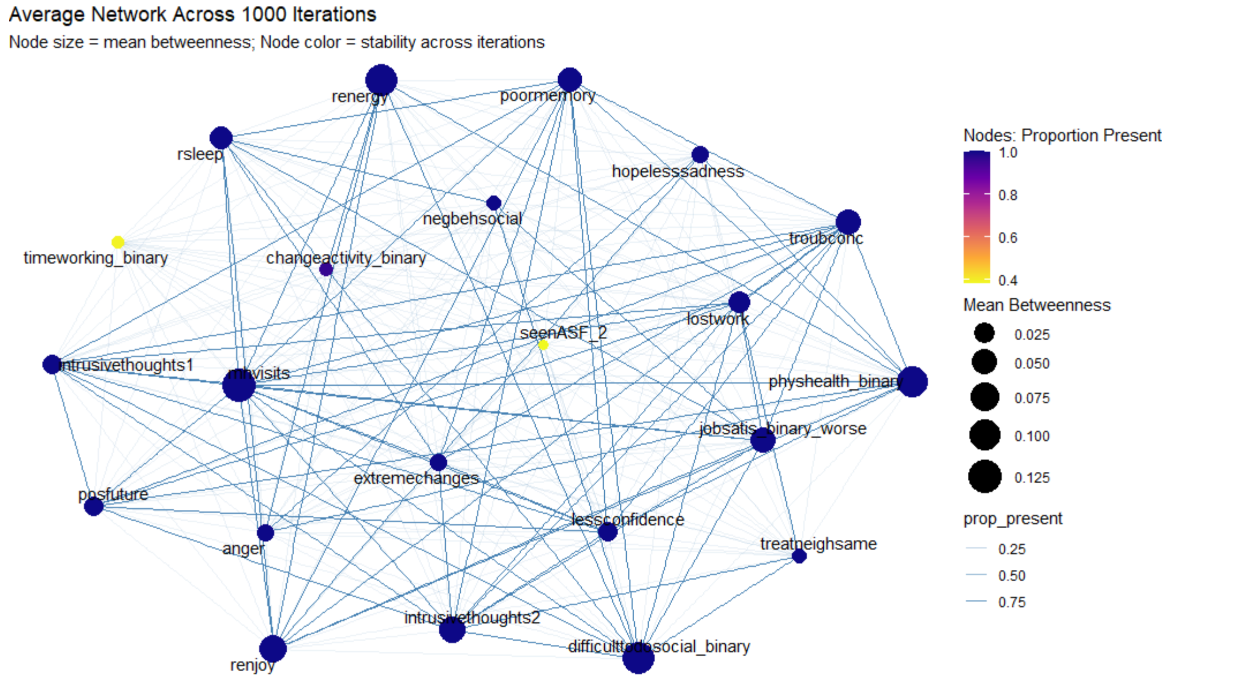

Average OPA Network with Simulated 10% Randomness in OPA Values

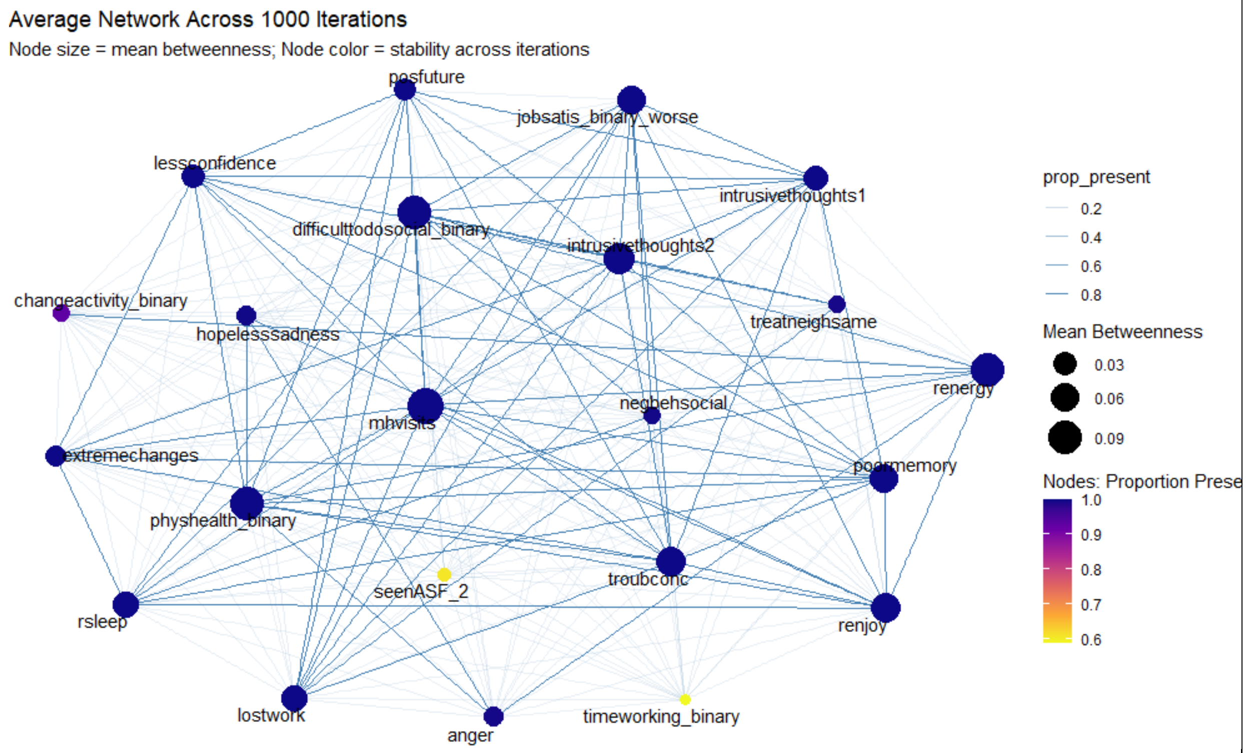

Average OPA Network with Simulated 20% Randomness in OPA Values

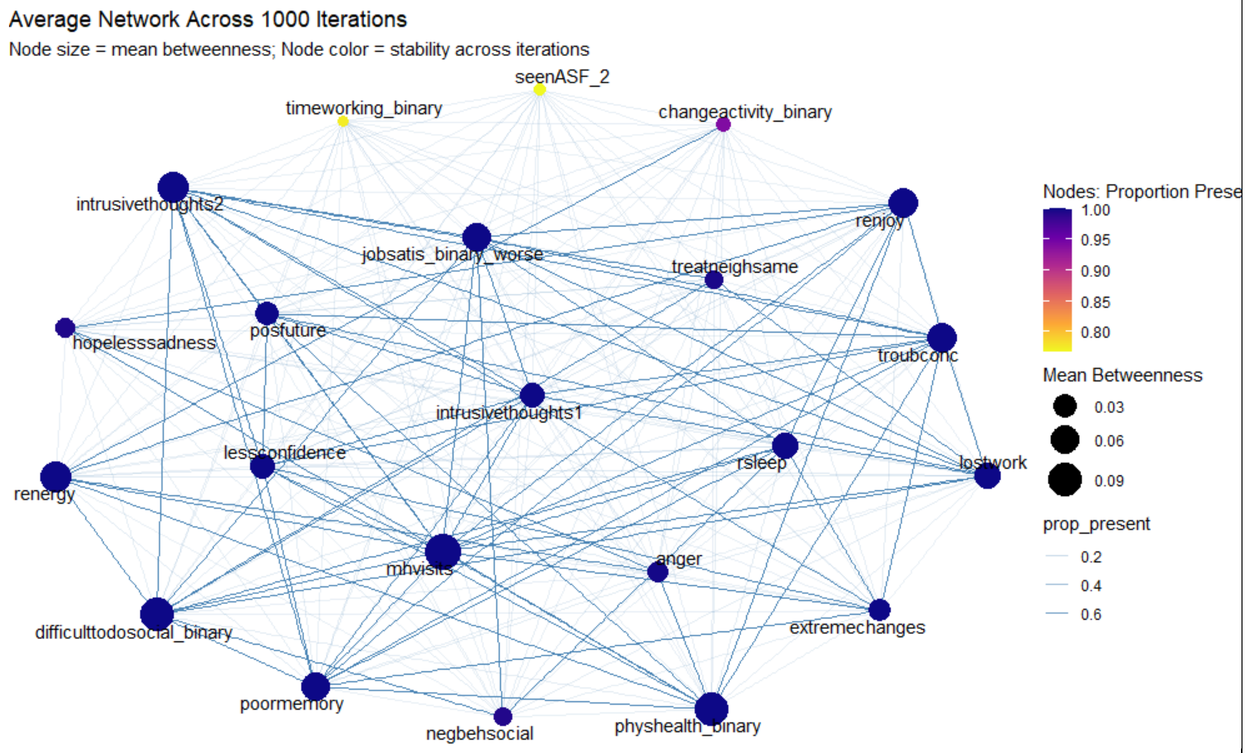

Average OPA Network with Simulated 30% Randomness in OPA Values

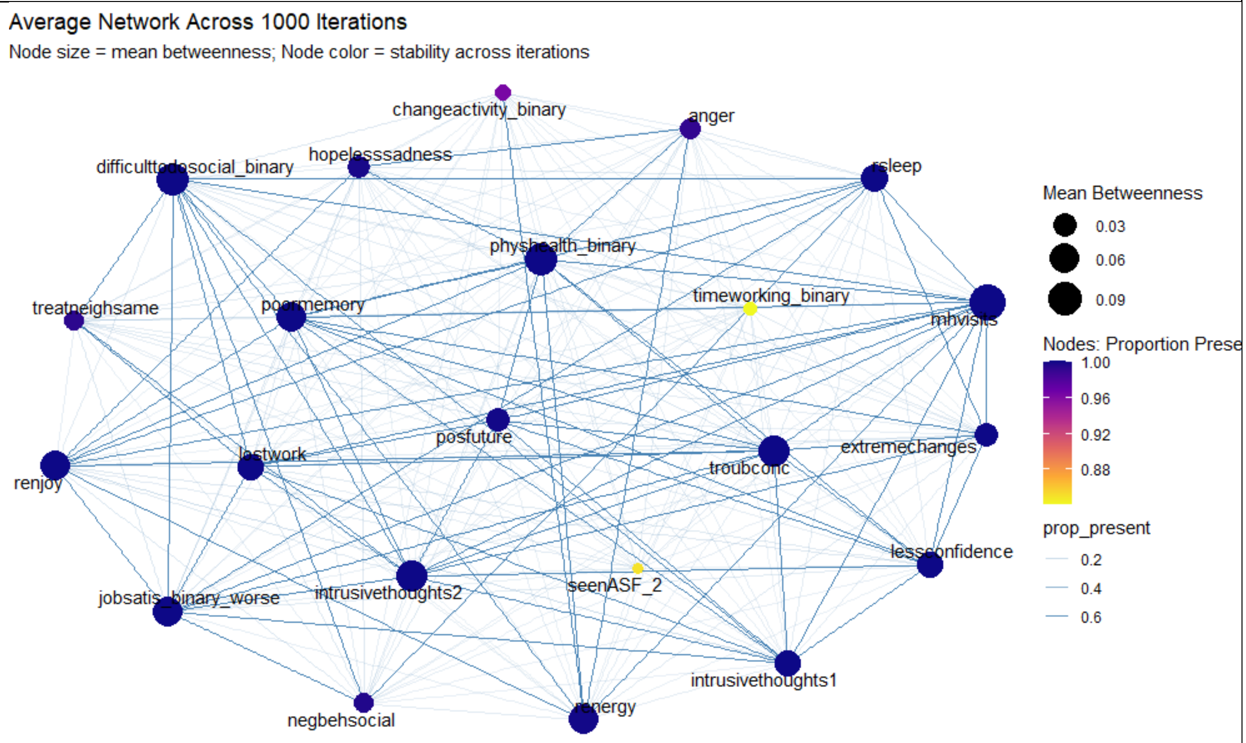

Average OPA Network with Simulated 40% Randomness in OPA Values

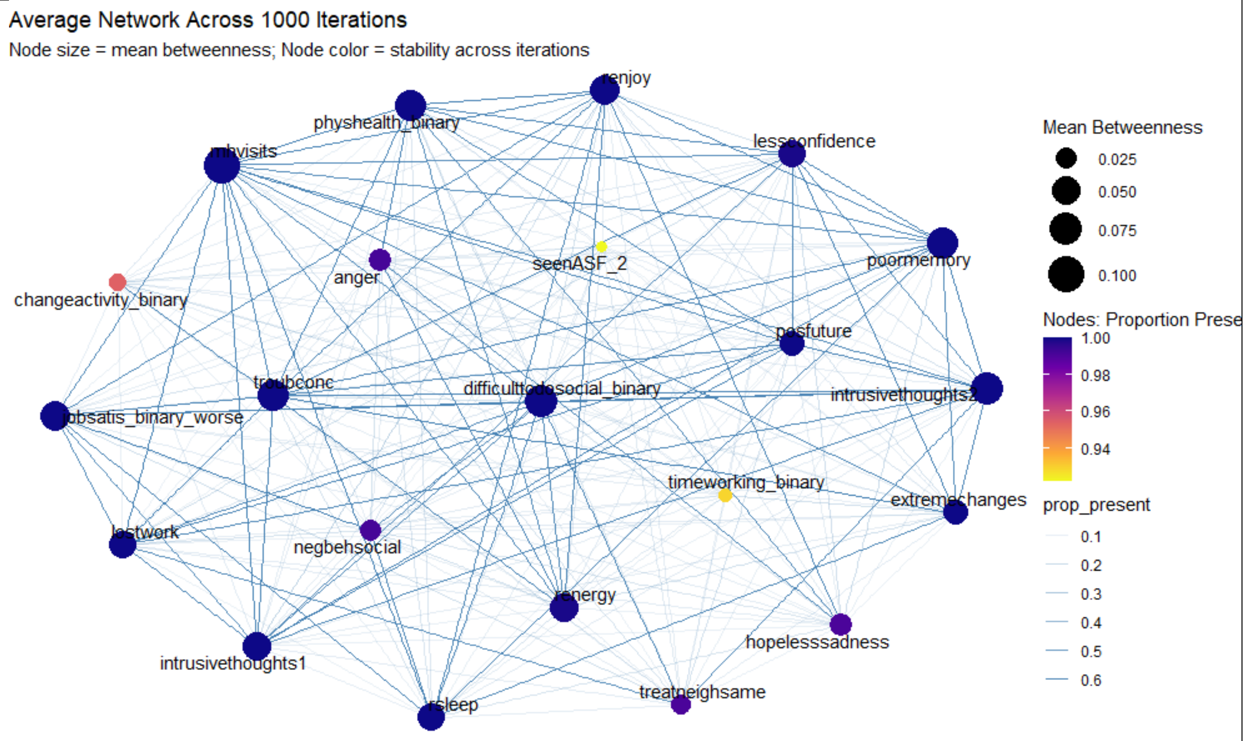

Average OPA Network with Simulated 50% Randomness in OPA Values

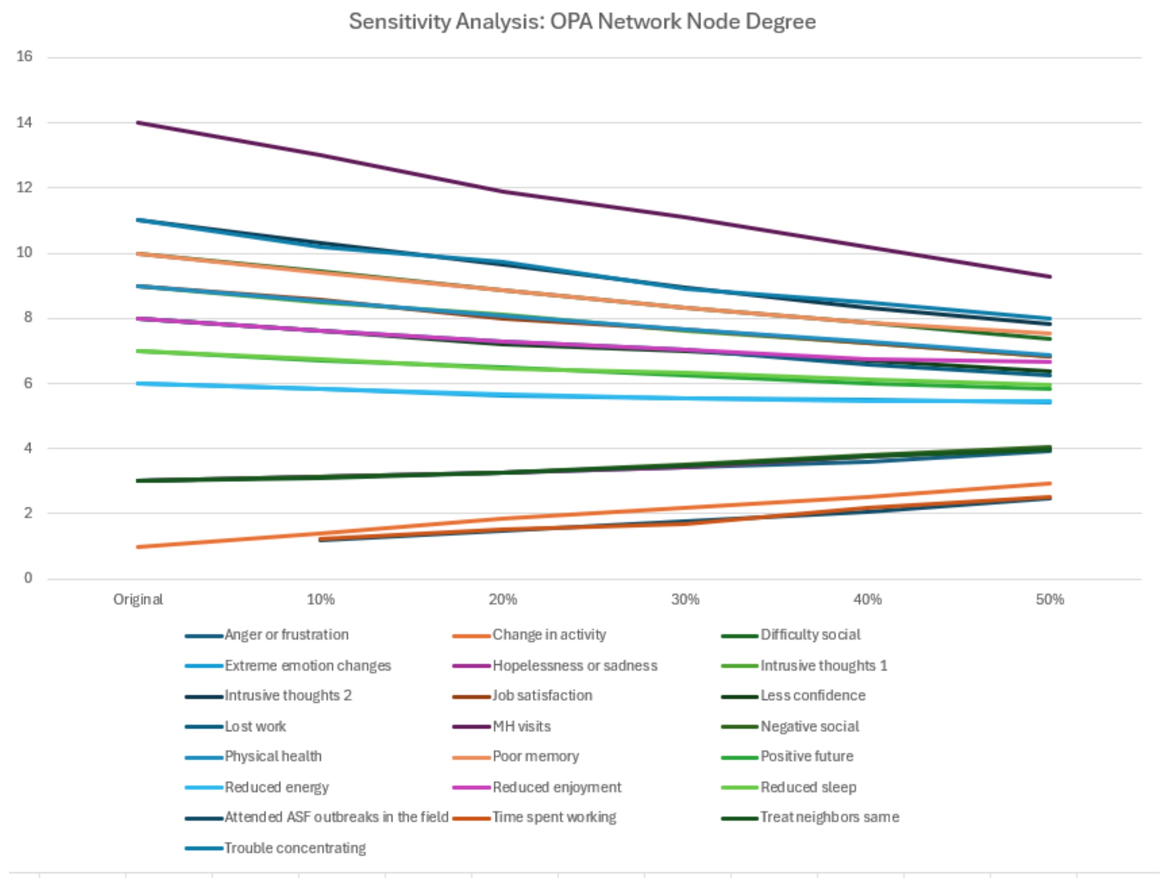

Mean value of node degree within OPA networks (Far left =Original network, then simulated networks with increasing randomness from left to right).

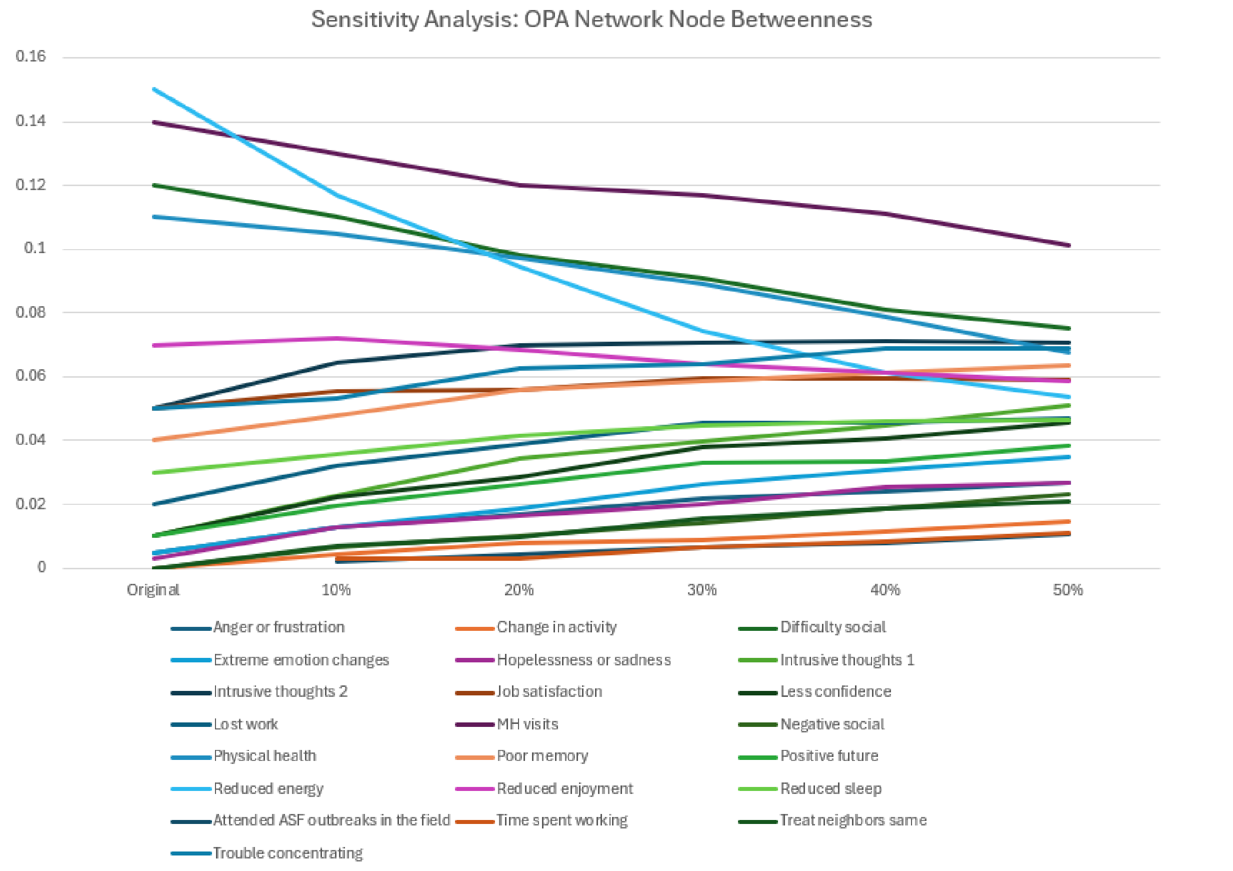

Mean value of node betweenness within OPA networks (Far left =Original network, then simulated networks with increasing randomness from left to right).

PPA Network

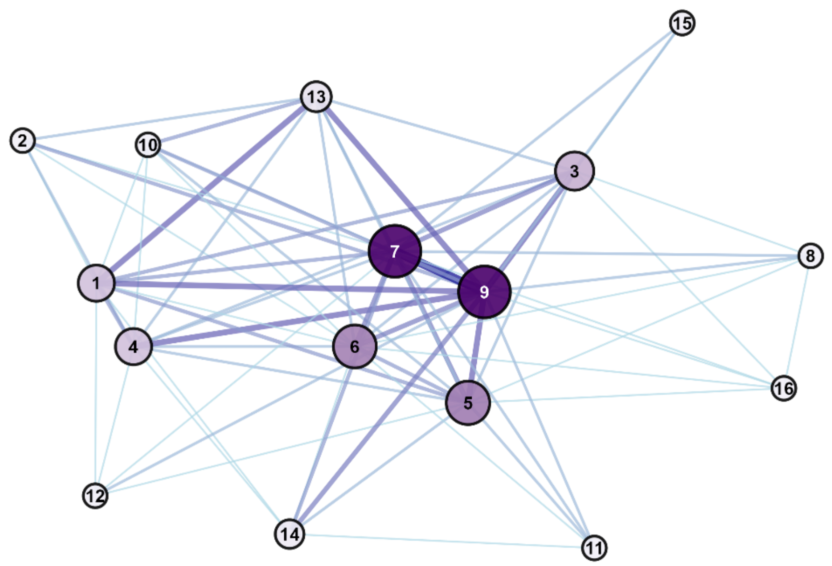

Number	Name
1	Saw field ASF
2	Change in activity
3	Physical health
4	Reduced energy
5	Reduced sleep
6	Reduced enjoyment
7	Hopelessness or sadness
8	Poor memory
9	Anger or frustration
10	Extreme emotion changes
11	Job satisfaction
12	Difficulty doing social
13	Time working
14	Experienced negative social
15	Less confidence
16	MH visits

Original Network (Figure 2b from main manuscript)

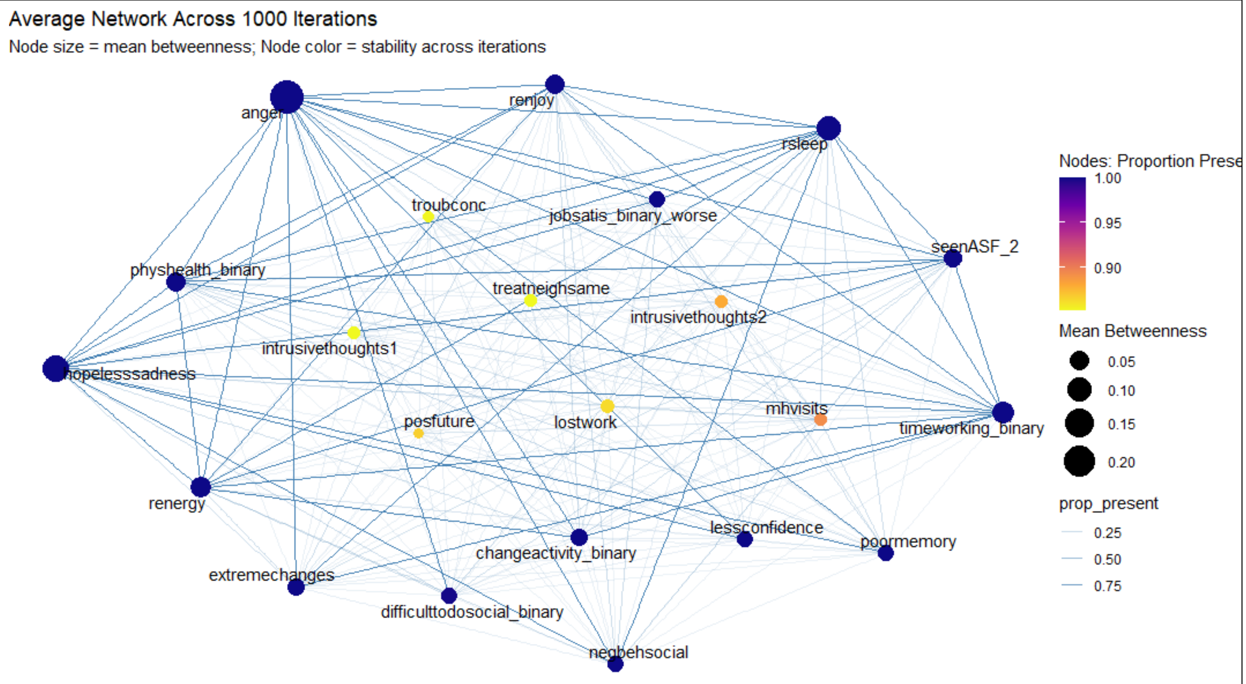

Average PPA Network with Simulated 10% Randomness in PPA Values

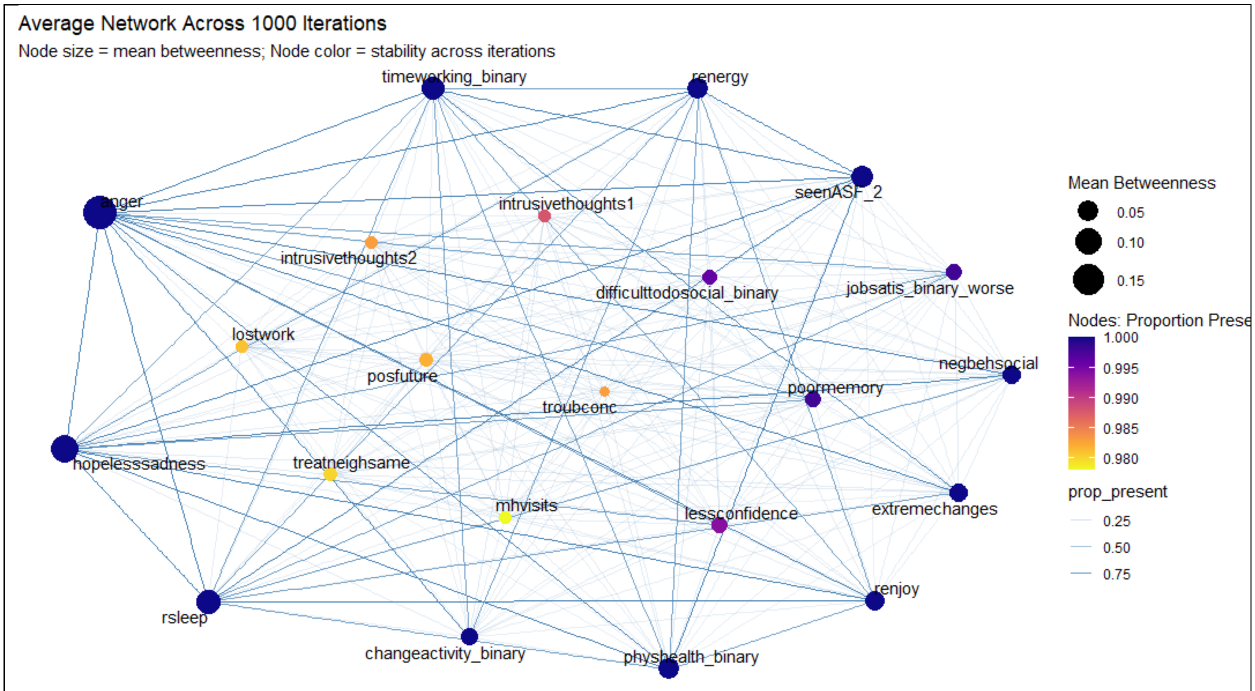

Average PPA Network with Simulated 20% Randomness in PPA Values

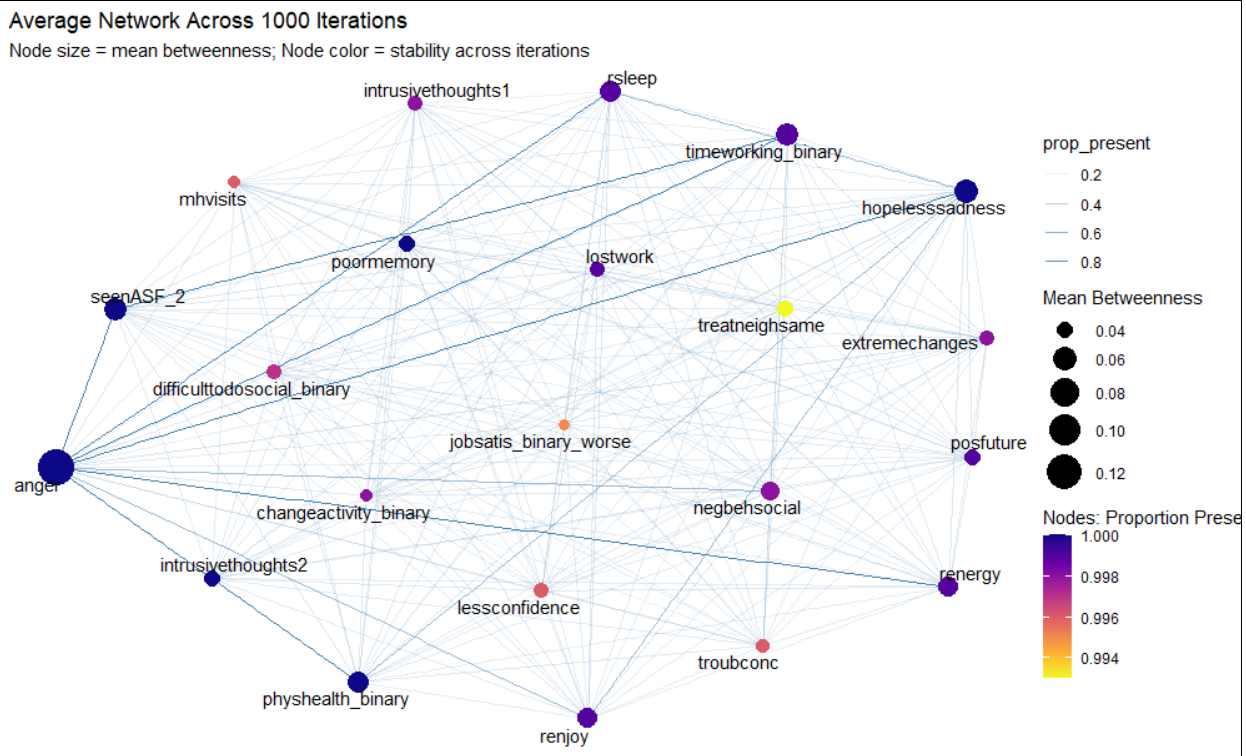

Average PPA Network with Simulated 30% Randomness in PPA Values

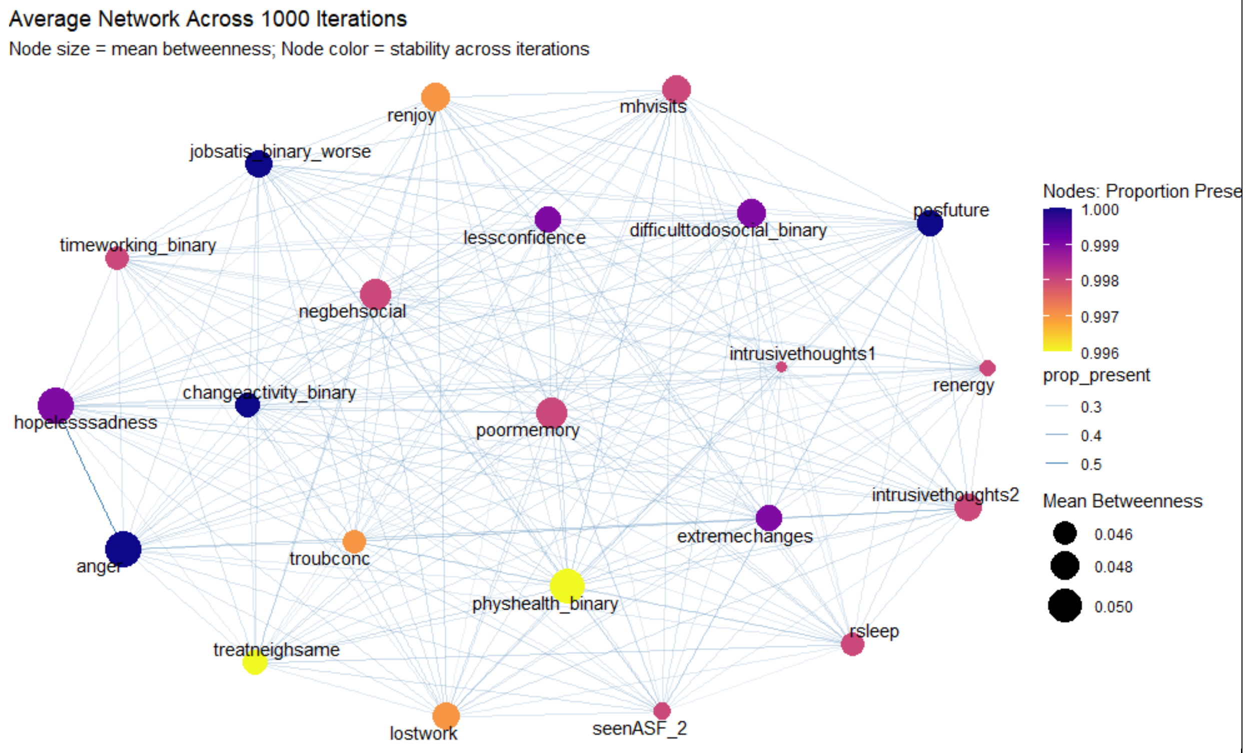

Average PPA Network with Simulated 40% Randomness in PPA Values

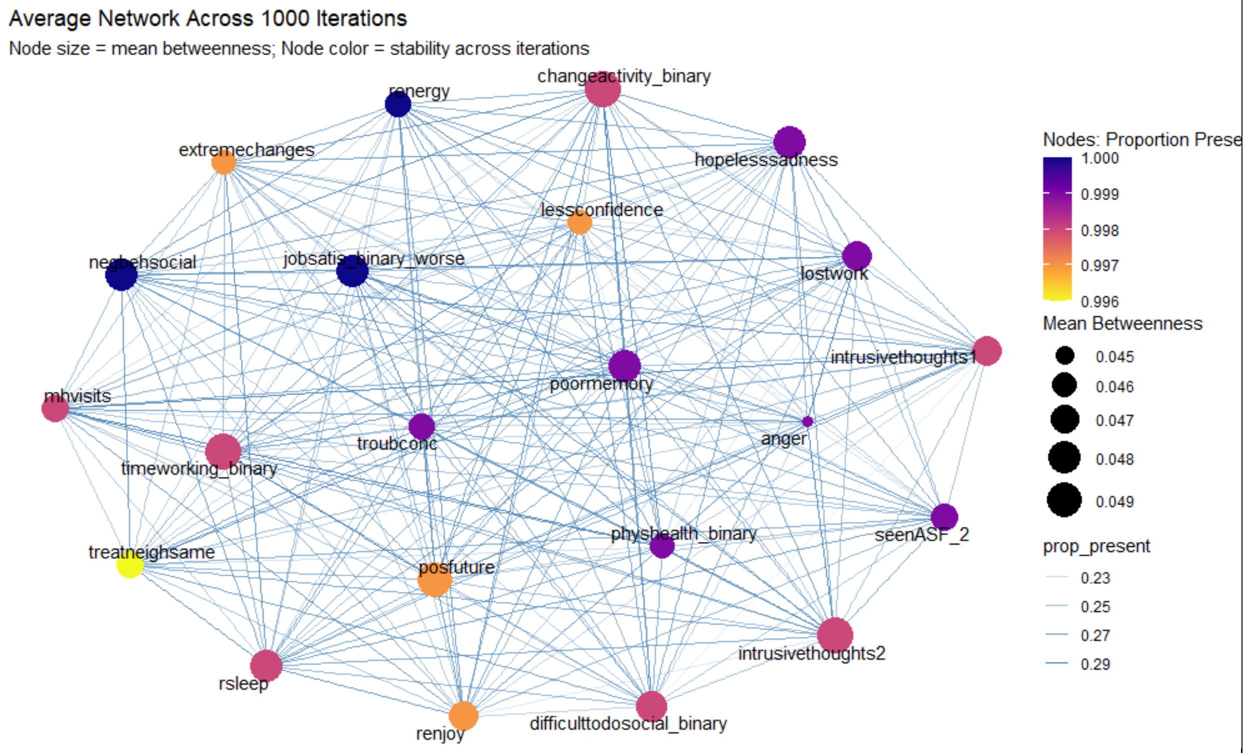

Average PPA Network with Simulated 50% Randomness in PPA Values

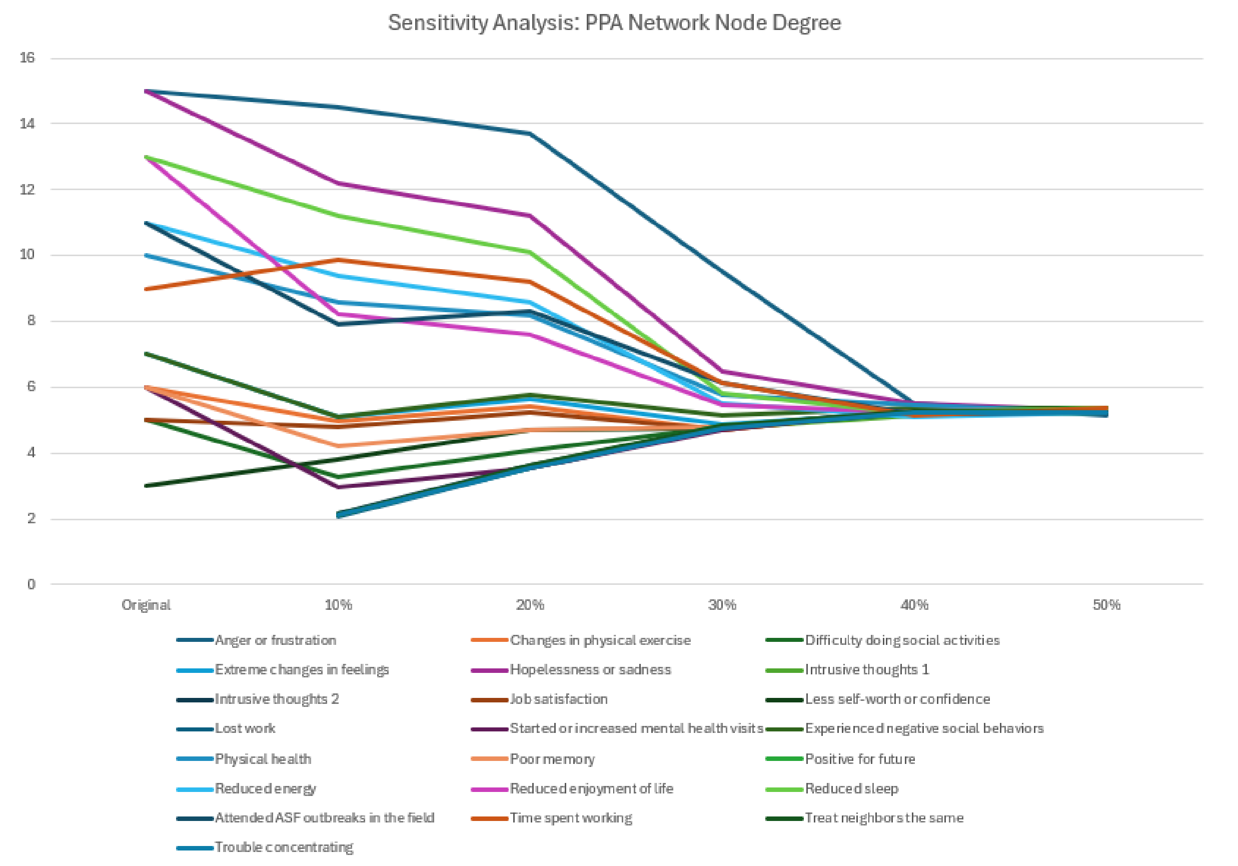

Mean value of node degree within PPA networks (Far left =Original network, then simulated networks with increasing randomness from left to right).

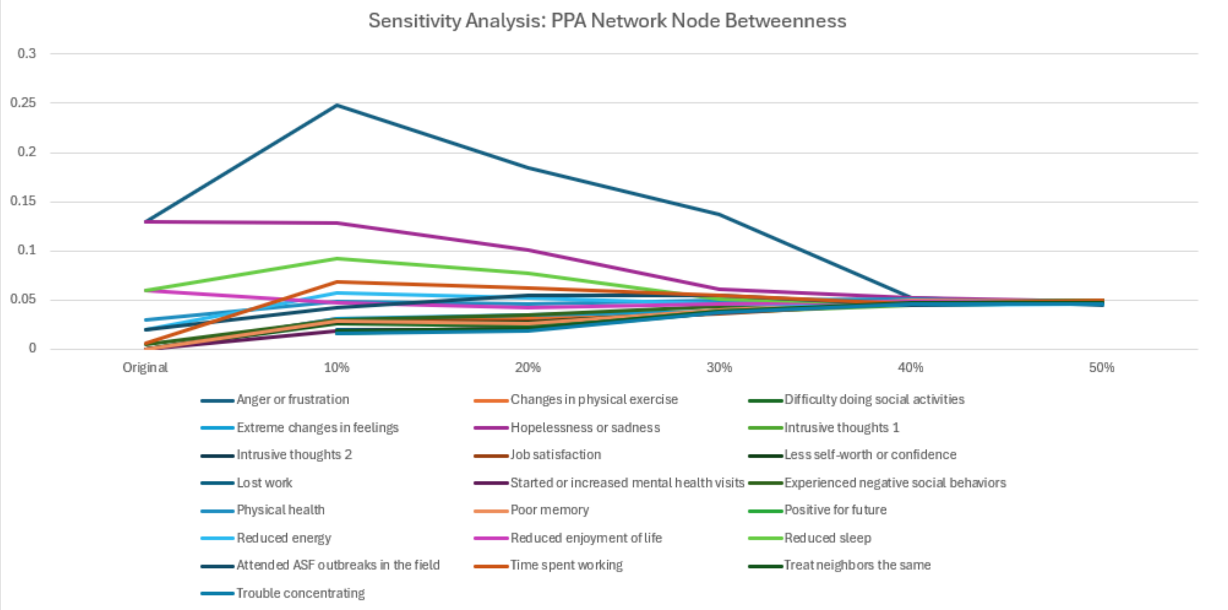

Mean value of node betweenness within PPA networks (Far left =Original network, then simulated networks with increasing randomness from left to right).
