# Supplementary material for: Impact of African swine fever emergency on the mental health of first responders in the Dominican Republic
Source: PLoS One. 2026 Feb 3;21(2):e0342159. doi: 10.1371/journal.pone.0342159 (PMC12867258; doi:10.1371/journal.pone.0342159)
Supplement: S1 Table — (PDF) [file pone.0342159.s005.pdf]

Supplementary Table 1. Dichotomization and leveling of variables for network analysis.

Variable	High Level (1)	Low Level (2)
Attended ASF Outbreaks	Yes	No
Time spent working	Much more, Somewhat more	About the same, Somewhat less, Much less
Job satisfaction	Much worse, Somewhat worse	About the same, Somewhat better, Much better
Physical health or emotional problems that caused difficulty doing social activities	Yes	No, No Change
Change in physical exercise	More	About the same, More
Physical health	Worse	Better, No Change
Positive about the future	No	Yes
Reduced energy	Yes	No
Reduced sleep	Yes	No
Reduced enjoyment of life	Yes	No
Hopelessness and sadness	Yes	No
Trouble concentrating	Yes	No

Poor memory	Yes	No
Anger	Yes	No
Extreme changes in emotions	Yes	No
Less confidence	Yes	No
Intrusive thoughts about death or dying	Yes	No
Intrusive thoughts that family or community would be improved if gone	Yes	No
Started mental health visits	Yes	No
Treat neighbors the same	No	Yes
Experienced negative social behaviors	Yes	No
Lost work	Yes	No
