# Supplementary material for: Impact of African swine fever emergency on the mental health of first responders in the Dominican Republic
Source: PLoS One. 2026 Feb 3;21(2):e0342159. doi: 10.1371/journal.pone.0342159 (PMC12867258; doi:10.1371/journal.pone.0342159)
Supplement: S2 Table — (PDF) [file pone.0342159.s006.pdf]

Supplementary Table 2. Global-level network measurements of High OPA and High PPA networks.

**Measurement**	**High OPA Network**	**High PPA Network**
Nodes	20	16
Edges	73	71
Edge density	0.384	0.592
Average degree	7.3 (range: 1-14)	8.9 (range: 3-15)
Global Transitivity (Clustering Coefficient)	0.598	0.697
Diameter	4	2
Mean Distance	1.79	1.41
Degree Centralization	0.392	0.467
Betweenness Centralization	0.108	0.104
Closeness Centralization	0.47	0.588
Global Efficiency	0.663	0.796
